# Frost resistance in alpine woody plants

**DOI:** 10.3389/fpls.2014.00654

**Published:** 2014-12-01

**Authors:** Gilbert Neuner

**Affiliations:** Unit Functional Plant Biology, Institute of Botany, University of InnsbruckInnsbruck, Austria

**Keywords:** ice susceptibility, freezing avoidance, frost dehardening, frost hardening, supercooling

## Abstract

This report provides a brief review of key findings related to frost resistance in alpine woody plant species, summarizes data on their frost resistance, highlights the importance of freeze avoidance mechanisms, and indicates areas of future research. Freezing temperatures are possible throughout the whole growing period in the alpine life zone. Frost severity, comprised of both intensity and duration, becomes greater with increasing elevation and, there is also a greater probability, that small statured woody plants, may be insulated by snow cover. Several frost survival mechanisms have evolved in woody alpine plants in response to these environmental conditions. Examples of tolerance to extracellular freezing and freeze dehydration, life cycles that allow species to escape frost, and freeze avoidance mechanisms can all be found. Despite their specific adaption to the alpine environment, frost damage can occur in spring, while all alpine woody plants have a low risk of frost damage in winter. Experimental evidence indicates that premature deacclimation in *Pinus cembra* in the spring, and a limited ability of many species of alpine woody shrubs to rapidly reacclimate when they lose snow cover, resulting in reduced levels of frost resistance in the spring, may be particularly critical under the projected changes in climate. In this review, frost resistance and specific frost survival mechanisms of different organs (leaves, stems, vegetative and reproductive over-wintering buds, flowers, and fruits) and tissues are compared. The seasonal dynamics of frost resistance of leaves of trees, as opposed to woody shrubs, is also discussed. The ability of some tissues and organs to avoid freezing by supercooling, as visualized by high resolution infrared thermography, are also provided. Collectively, the report provides a review of the complex and diverse ways that woody plants survive in the frost dominated environment of the alpine life zone.

## INTRODUCTION

Freezing temperatures are a major environmental stress factor in the alpine life zone and in the treeline ecotone. Woody plants in these areas are frequently exposed to conditions that can result in frost damage. Frost resistance of tree species present at the alpine treeline in temperate climates has been addressed several times in prior reviews (Tranquillini, 1979; Neuner, 2007; Körner, 2012). Less information is available for other woody alpine species that do not grow to the height of trees. Additionally, frost resistance has often been exclusively studied in leaves and detailed seasonal records have been reported. Frost survival of an individual, however, will also depend, if not more so, on the survival of other organs, such as bud and stem tissues. The degree of frost resistance and the underlying frost survival mechanisms of these essential organs are, in many cases, still unknown. The same is true for the effect of frost on reproductive tissues, which can have a dramatic impact on overall reproductive success. Improvements in imaging methods to study the freezing processes in plants, such as infrared differential thermal analysis (IDTA; Hacker and Neuner, 2007, 2008; Hacker et al., 2008; Neuner et al., 2010; Kuprian et al., 2014), has led to significant advances in our current understanding of ice propagation, ice barriers, and supercooling in plants. IDTA can provide more precise information on tissue-specific mechanisms of frost survival and on the basis of frost damage. Additionally, the development of field portable frost chambers (Neuner et al., 1999b; Taschler and Neuner, 2004; Buchner and Neuner, 2009), provides the ability to simulate night frosts and study recovery and recuperation under natural conditions, even though the use of the chambers may not be able to mimic a true radiation frost (Frederiks et al., 2012). These combined approaches can provide results that are more ecologically relevant for the prediction of future scenarios of plant survival and distribution. This may become even more important in view of global warming, as alpine environments are categorized as highly vulnerable ecosystems under climate change projections (Theurillat and Guisan, 2001; Byars et al., 2007).

Frost-induced drought and photodamage, typical parameters of winter stress (Neuner et al., 1999a) that often occur along with freezing stress, are not addressed in this review, although they may also be critically important for winter survival of woody species. Processes related to cold acclimation have been reviewed by Pearce (1999), Thomashow (1999), and Xin and Browse (2000) are also not the primary focus of this review.

## FREEZING TEMPERATURES IN THE TEMPERATE ALPINE ZONE

**FIGURE 1:**
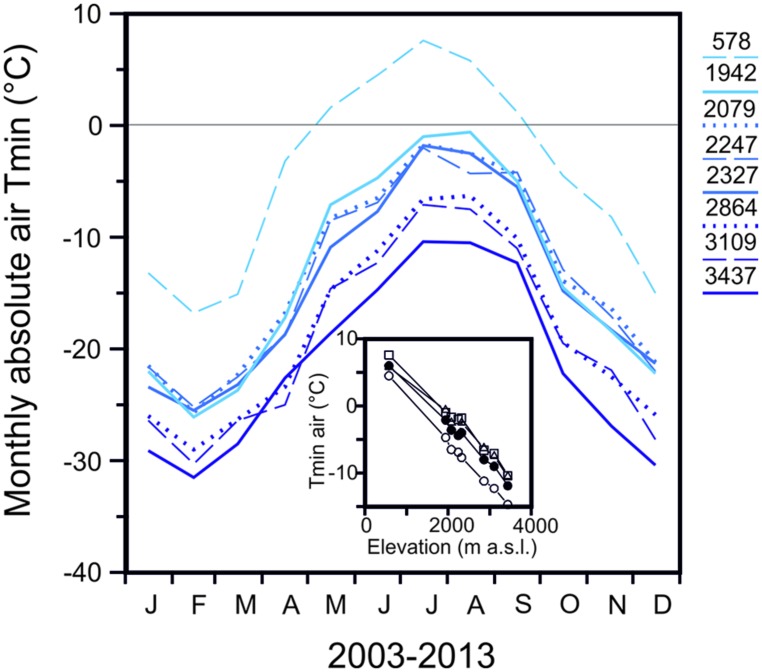
**Monthly absolute air temperature minima obtained for the period 2003–2013 from records of meteorological stations located at the indicated elevations in the Central European Alps of Austria [Innsbruck-University (578 m), Obergurgl (1942 m), Patscherkofel (2247 m), Galzig (2079 m), Ischgl-Idalpe (2327 m), Pitztaler Gletscher (2864 m), Sonnblick (3109 m), Brunnenkogel (3437 m)].** Inset: Reduction of absolute air temperature minimum with increasing elevation during the snow free period in (○) June, (△) July, (□) August and (●) the summer mean (all data from ZAMG, Austria).

The temperate alpine life zone extends from treeline at approximately 2000 m (spread over several 100 m of altitude) up to the altitudinal limit of closed vegetation, around 3480 m (Grabherr et al., 1995; Körner, 2003). The ultimate limit of individual higher plant life in the European Alps was reported to be at 4507 m (Körner, 2011). Woody species, of course, have a lower upper distribution boundary, which is around 3000 m (*Salix herbacea,* Landolt, 1992), than herbaceous plants. Freezing temperatures can occur throughout the whole year in the temperate alpine zone and their severity increases significantly with elevation. The seasonal change in monthly minimum air temperatures (2003–2013) of seven meteorological stations in the Austrian Central Alps is shown in comparison to a low land site (Innsbruck 578 m; **Figure 1**) in order to highlight the characteristics of the alpine life zone with respect to freezing temperatures. The meteorological stations within the alpine life zone are located along an elevational gradient of approximately 1500 m. While a considerable frost free period is evident (May till September) at the lowland site, a characteristic of the alpine life zone is the lack of a frost free period, as freezing temperature can occur virtually throughout the whole year. At an elevation of 1942 m, freezing temperatures (-4.7°C in June and -1.0°C, July/August) can occur during the summer months, while at 3437 m, temperatures can drop substantially lower (-14.7 June and -10.5°C July/August; **Figure 1** inset). In midwinter, air temperatures can drop to -26.1 (February) at 1942 m and to -31.5°C at 3437 m. The adiabatic lapse rate from these air temperature minima is -0.7 K^.^100 m^-1^. In addition to the increase in frost severity with elevation, the frequency of freezing events also rises significantly with increasing elevation within the alpine life zone (Taschler and Neuner, 2004; Larcher and Wagner, 2009; Neuner and Hacker, 2012).

## PLANT MINIMUM TEMPERATURES

It is possible for the temperature of plant material to be lower than air temperature. Specifically, the net radiation balance of leaves can become negative during clear, calm nights due to long-wave radiative heat loss (Stefan-Boltzmann-law) which cools the leaves below air temperature. This is the basis for dew or surface ice formation but can also result in the freezing of plant tissues despite an air temperature above 0°C. Leaves of conifers were found to be up to 9.3 K colder than air temperature at treeline (Tranquillini, 1958, 3–8 K: Jordan and Smith, 1994). Air temperature may exhibit an extreme drop down to -37.4°C (Mt. Sonnblick, 3105 m, 1.1.1905, Austria; Central Institute for Meteorology and Geodynamics, Austria) in alpine areas of Austria during the winter. Winter temperature minima the last ten years (2003–2013) have ranged between -26.1°C (1942 m) and -31.5°C (3437 m). Based on these data, leaf temperatures down to approximately -35 to -46°C may occur in exposed plants during clear and calm winter nights (Neuner, 2007). Radiative cooling conditions in June may account for leaf temperatures down to approximately -14 to -24°C in July and down to approximately -10 to -20°C in August.

In summer, mean leaf temperature minima decrease with increasing elevation at a rate of 0.4 K.100m^-1^ (Neuner and Hacker, 2012) which is less than the adiabatic lapse rate of air (0.7 K.100m^-1^). The difference may be explained by the change in growth forms and snow protection of plants during cold spells in summer. Bioclimate temperatures lower than 0°C during the snow free period were found to occur at a frequency of 20% at the treeline (1950 m), while at 3450 m night frost frequency increased to 69% (Larcher and Wagner, 2009).

In addition to the severity and frequency of freezing temperatures, cooling and warming rates also appear to be important for frost survival of woody plants. Natural cooling rates below 0°C in leaves of alpine plants did not exceed -2K.h^-1^ (mean rate: -0.3 K.h^-1^; Neuner and Buchner, 2012). Exceptionally more rapid cooling rates, however, cannot be fully excluded, as reported by Körner (2012). In northern Sweden, temperature drops below 0°C of up to -15 K within minutes were recorded at the upper border of cold air drained during a temperature inversion. This rapid temperature drop resulted in the mortality of *Betula pubescens* buds that otherwise would have survived exposure to freezing temperatures down to -70°C under moderate cooling rates (Körner, 2012).

**FIGURE 2:**
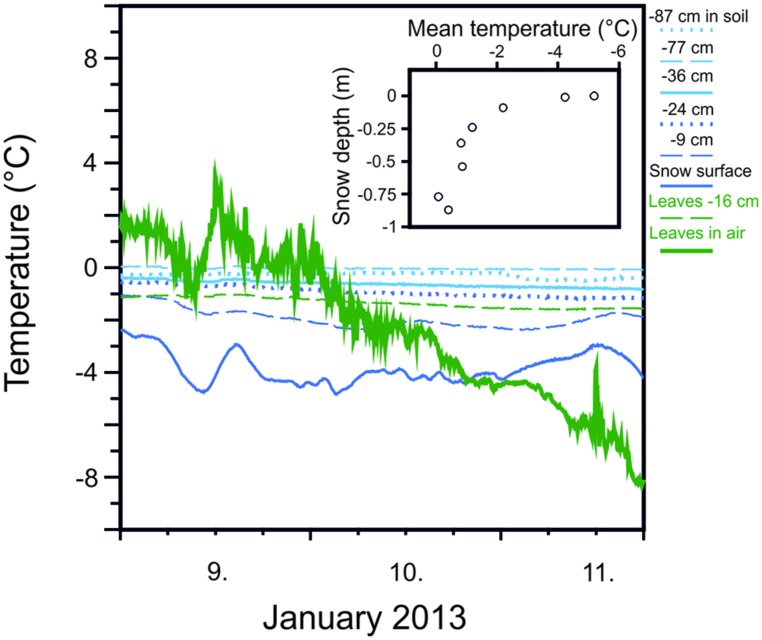
**Mitigation of minimum temperatures with increasing snow depth measured at various depths within the winter snow cover (77 cm deep) and in soil (–10 cm) during the course of three days in January, 2013 at 1950 m on Mt. Patscherkofel.** Snow temperature is compared to leaf temperatures recorded for leaves of *Rhododendron ferrugineum* that were either exposed without snow cover (green solid line) or covered by snow (–16 cm). Inset: Increasing mean temperature (9, 10, and 11 January, 2013) in snow with increasing depth within a snow cover of 77 cm (Buchner and Neuner, unpublished).

Snow cover significantly mitigates temperature minima and the rate of temperature change to which plants are exposed. Important factors contributing to of the degree of thermal insulation are the thickness of the snow pack (**Figure 2**) and its consistency (Kuhn, 2012). While protective effects of snow cover have long been recognized (Sakai and Larcher, 1987), thermal insulation by snow may also be crucially important for protecting plants from low freezing temperatures during episodes of freezing temperatures in summer (Larcher and Wagner, 2009; Ladinig et al., 2013). A certain degree of thermal insulation is possible by snow coverage for dwarf shrubs and young trees in the summer months but will be lost at a certain stage of tree development when a specific tree height is reached. The establishment of tree seedlings and young plants may even be favored at growing sites with sufficient snow coverage. On the other hand, being tall as a tree actually reduces the risk of frost injury during summer radiation frosts compared to small statured woody plants because radiative freezing during clear nights is negatively correlated with tree height (Körner, 2012).

## ORGAN-SPECIFIC RISK OF FROST DAMAGE IN WOODY ALPINE PLANTS

**FIGURE 3:**
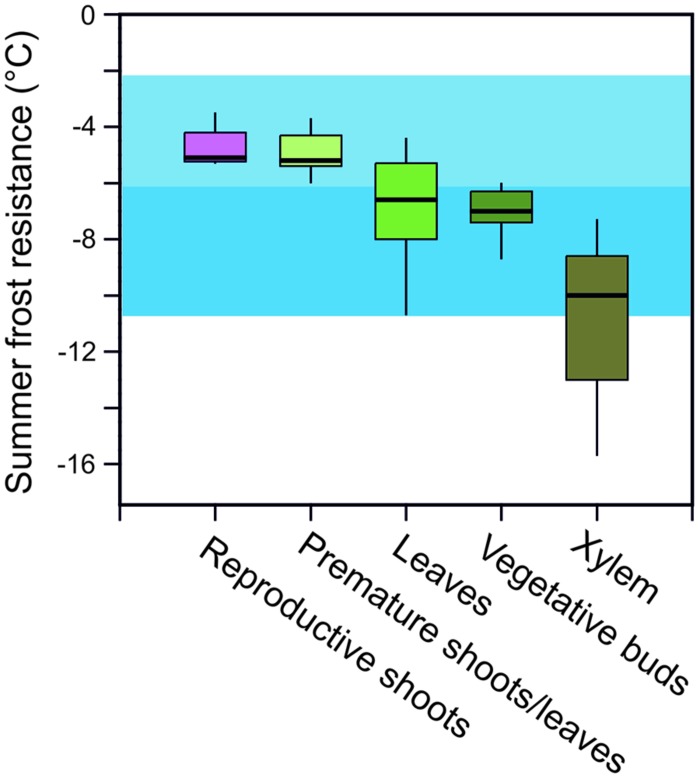
**Frost resistance (LT_**i**_/LT_**10**_; °C) of different organs of woody alpine plant species listed in **Table 1** assessed during summer [data compiled from: Sakai and Larcher (1987), Taschler and Neuner (2004), Ladinig et al. (2013), and Neuner et al. (unpublished)].** Air temperature minima in June (blue horizontal bar) and July and August (light blue horizontal bar) as recorded at the upper distribution boundary of the species (2100–2800 m) in the years 2003–2013 (see legend to **Figure 1**). LT_i_/LT_10_ = freezing temperature causing initial and 10% frost damage. Box plots show the median, and the 25 and 75% quartiles, respectively, given by the lower and upper edges of boxes. Whiskers show the 95% confidence interval. Outliers are not shown.

In addition to minimum temperatures, the duration of a frost event and the rate of temperature change that can occur at specific parts of the year, play an essential role in determining the potential risk of frost injury to plants and the actual killing temperature of any specific plant species. Frost survival of individual woody plants is difficult to assess as frost resistance can differ greatly in various organs and tissues. For instance, the most frost susceptible organs in woody alpine plants in the summer are reproductive shoots (-4.6°C) followed by developing leaves (-5.0°C), fully expanded leaves (-6.6°C), vegetative buds (-7.3°C), and then xylem tissue (-10.8°C; **Figure 3**). While hardly any data exist for roots, they are commonly recognized as being the most susceptible to frost injury (Sakai and Larcher, 1987).

A survey of the frost resistance of different organs and tissues of 15 species of woody plants are presented in **Table 1**. The selected species comprise four trees, two tall shrubs, and nine dwarf shrubs that all have different vertical distributions in the Central European Alps.

**Table 1:** Characteristics of the surveyed species, after Landolt (1992).

Growth form	Plant species	Growth height (m)	Vertical distribution(ma.s.l.) in the Alps	Elevational belt
Trees	*Larix decidua*	<40	800–2,400	Submontane-alpine
	*Picea abies*	<60	600–2,100	Submontane-alpine
	*Pinus cembra*	<20	1,600–2,400	Alpine
	*Sorbus aucuparia*	10	600–2,400	Submontane-alpine
Tallshrubs	*Alnusalno-betula*	<3	1,000–2,300	Montane-alpine
	*Pinus mugo*	<2	1,000–2,400	Montane-alpine
Dwarfshrubs	*Arctostaphylosuva-ursi*	0.05–0.25	600–2,600	Submontane-alpine
	*Calluna vulgaris*	0.1–0.5	400–2,600	Submontane-alpine
	*Empetrum hermaphroditum*	0.1–0.2	1,700–2,600	Alpine
	*Juniperus nana*	<0.5	1,800–2,600	Alpine
	*Loiseleuria procumbens*	<0.05	1,800–2,800	Alpine
	*Rhododendron ferrugineum*	0.3–0.8	1,000–2,500	Montane-alpine
	*Vaccinium myrtillus*	0.05–0.5	300–2,600	Submontane-alpine
	*Vaccinium uliginosum*	0.1–0.2	1,500–2,700	alpine
	*Vaccinium vitis-idaea*	0.05–0.25	600–2,700	Submontane-alpine

## REPRODUCTIVE SHOOTS IN SUMMER

Various strategies have evolved in alpine plants to prevent frost damage to reproductive shoots. To date, the only alpine plant species found to actually exhibit freezing tolerance in its reproductive shoots is the nival herb *Ranunculus glacialis* (Ladinig et al., 2013). All other species have been reported to be freezing sensitive, i.e. exhibit injury if ice forms in their reproductive shoots (Ladinig et al., 2013). In contrast, fully developed vegetative parts of woody alpine plant species, such as stem, bark, and leaves can tolerate extracellular ice formation down to a species-specific low temperature during the summer months without damage (Taschler and Neuner, 2004). Once ice formation is initiated somewhere inside a vegetative part of a plant, it can propagate rapidly (up to 27 cm.s^-1^) throughout the vascular system in all plant parts colder than 0°C (Hacker and Neuner, 2007, 2008; Hacker et al., 2008).

Freezing sensitive reproductive shoots of some alpine species have evolved supercooling strategies aided by ice barriers in order to prevent frost injury. The ice barriers prevent the spread of ice from frozen plant parts into the supercooled organs or tissues. In cushion plants, the ice barriers are thermal rather than physical. The spread of ice from one flower stalk to the next is interrupted by the thermal insulation that exists inside the cushion portion of the plant from which the individual flower stalks arise, maintaining the cushion warmer than 0°C during the course of a night frost event (Hacker et al., 2011). Structural ice barriers have also been found in some species of woody plants (Workmaster et al., 1999; Carter et al., 2001; Kuprian et al., 2014). The specific nature of these physical barriers is currently under investigation. It appears that some unique aspect of the anatomical structure in the portion of the stem subtending the reproductive shoot prevents ice propagation into the shoot, allowing it to remain supercooled.

The probability of frost damage to reproductive shoots becomes greater and greater with increasing elevation and has been observed to occur on a recurrent basis in nature (Ladinig et al., 2013). Scarce information exists, however, about the frost resistance of reproductive tissues in woody alpine species (**Table 2**). The frost resistance of reproductive tissues of two woody alpine species (*Rhododendron ferrugineum* and *Loiseleuria procumbens*) was recently investigated (Ladinig et al., 2013). During anthesis and fruiting in the summer months, the reproductive shoots of these species were freezing sensitive and significantly less frost resistant than their ice tolerant vegetative shoots (Ladinig et al., 2013). Ice propagation into the reproductive shoots of *R. ferrugineum* via the vegetative shoot is not prevented by any ice barrier and any ice formation in the plant results in frost damage to reproductive tissues (Neuner and Hacker, 2012), which can occur at -3.3°C (Ladinig et al., 2013). In contrast, anatomical ice barriers efficiently prevent ice intrusion into the supercooled reproductive shoots of *L. procumbens* and other woody species in the summer months (Kuprian et al., 2014). This allows reproductive shoots to remain in a supercooled state during a night frost and survive completely undamaged despite freezing of the vegetative parts. While vegetative parts froze between -4.7 and -5.7°C, reproductive shoots were able to supercool down to a range of -7.2°C to -18.2°C, and in some cases even below -22°C (Kuprian et al., 2014). This mechanism of freeze avoidance has been shown to be effective in preventing frost injury during various stages of reproductive development (bud, anthesis, and fruit) throughout the alpine summer. Supercooling was 100% effective in preventing frost damage to reproductive shoots in the woody shrub species *Calluna vulgaris* and *Empetrum hermaphroditum*, while the ice barrier failed to prevent ice entrance in *L. procumbens* in 13% of the total observations (Kuprian et al., 2014). The frost survival mechanism of reproductive shoots in summer months in other alpine woody species is currently unknown.

**Table 2:** The mechanism of frost survival in different plant parts within the same plant species and maximum frost resistance attained under natural acclimating conditions.

Growth form	Plant species	Reproductive shoots summer	Reproductive buds winter	Vegetative buds summer	Vegetative buds winter	Immature leaves	Mature leaves Summer	Mature Leaves winter	Stem summer	Stem winter
Trees	*Larix decidua*	–	–	Sc^4^ (–6.4)^4^	Sc^4^ (–41.4)^4^	Is (–6.3)^5^	Ft (–8.0)^6^	–		?–70^7^
	*Picea abies*	–	–	Sc^4^ (–10.3)^4^	Sc^4^ (–31.0)^4^	Is (–4.4)^5^	Ft (–5.1)^6^	Ft (–43.4)^4^	?–10^4^	
	*Pinus cembra*	–	–	–	Ft^4^ (–63.3°C)^8^	Is (–4.8)^5^	Ft (–6.7)^6^	Ft (–58.5)^8^	?–10^4^	
	*Sorbus aucuparia*	–	–	–	–	Is (–5.5)^5^	Ft (–6.8)^6^	–		
Tall shrubs	*Alnusalno-betula*	–	–	?–6.6^4^	?–40.8^4^		Ft (–4.8)^6^	–		
	*Pinus mugo*	–	–	–	Ft^4^ (–63.6)^8^		Ft (–8.3)^4^	Ft (–54.0)^4^		
Dwarf shrubs	*Arctostaphylosuva-ursi*	Sc (f)^2^	Sc^4^ (–18,5)^4^	–	?–30^7^		?–7^7^	Ft (–41.6)^4^		–30^7^ Sc^10^–35^10^
	*Calluna vulgaris*	Sc (b, a, f)^1^	–	–	?–30^7^		Ft (–8.9)^6^	Ft (–35.5)^7^		?–30^7^
	*Empetrum hermaphroditum*	Sc (b, a, f)^1^	–	–	–		–	Ft (–30)^7^		?–30^7^
	*Juniperus nana*	–	–	–	–		Ft (–9.0)^6^			
	*Loiseleuria procumbens*	Sc (b, a, f)^1^	Sc^1^ (–20)^7^	–7.3^9^	?–50^7^	?–5.7^9^	Ft (–6.6)^6^	Ft (–44.7)^4^	?–7^7^	?–40/–60^7^
	*Rhododendron ferrugineum*	Is^3^ (–3.3)^9^	Sc^3^ (–25)^7^	–	Sc^4^ (–30)^7^	Is (–3.4)^5^	Ft (–4.7)^6^	Ft (–25)^7^	?–16^4^	?–30^7^
	*Vaccinium myrtillus*		–	–	Sc^4^; **Figure 5** (–35)^7^		Ft (–4.1)^6^			?–35^7^
	*Vaccinium uliginosum*	–	–	–	?–40^7^		Ft (–5.6)^6^			?–50^7^
	*Vaccinium vitis-idaea*	–	Sc^4^; **Figure 4** (–30)^7^	–	?–30^7^		Ft (–5.5)^6^	?–70^7^		?–30^7^

*Sc, supercooling; Ft, freezing tolerance; Is, ice susceptible (freezing sensitive); ?, unknown mechanism; b, bud; a, anthesis; f, fruiting stages.*

*^1^Kuprian et al., 2014, ^*2*^Wisniewski et al., 2014, ^*3*^Neuner and Hacker, 2012, ^*4*^LT_*50*_ (Neuner et al., unpublished), ^*5*^LT_i_ (Taschler et al., 2004), ^*6*^LT_i_ (Taschler and Neuner, 2004), ^*7*^LT_*0*_ (Sakai and Larcher, 1987), ^*8*^LT_*50*_ (Buchner and Neuner, 2011), ^*9*^LT_*10*_ (Ladinig et al., 2013), ^*10*^LT_*50*_ (Becwar et al., 1981).*

## REPRODUCTIVE OVERWINTERING BUDS

**FIGURE 4:**
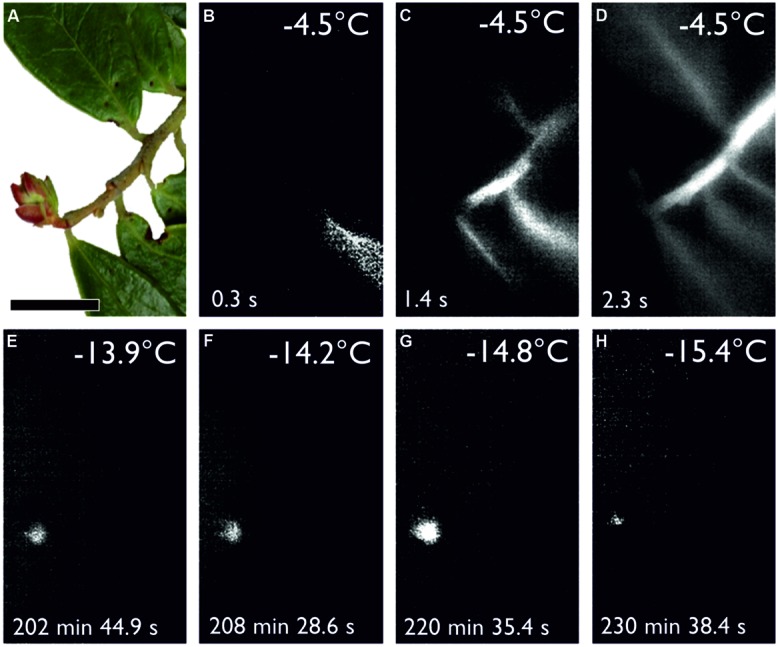
**(A)** Digital image of a shoot bearing a reproductive winter bud of *Vaccinium vitis-idaea* sampled on Mt. Patscherkofel (2010 m a.s.l.) during snow melt on 12.5.2014 before exposure to a controlled freezing treatment with a cooling rate of 3 K/h from +2°C down to -22.0°C (horizontal black bar width = 1 cm). **(B–D)** IDTA images show initial ice nucleation in a single leaf at -4.5°C. Ice propagates unhindered throughout the leaf into the vegetative shoot and the attached leaves but not into the reproductive bud. **(E–H)** The sequence of IDTA images shows the occurrence of independent freezing events in the reproductive bud at temperatures ranging from -13.9 to -15.4°C, which are lower than the temperature at which ice nucleation occurred in the vegetative part of the shoot. Freezing is visualized by a brightening in the image, while unfrozen areas remain black. The time elapsed after the occurrence of ice nucleation in the leaf is indicated in the bottom left corner of each image (Kuprian and Neuner, unpublished).

Overwintering reproductive buds of many angiosperm woody plants survive freezing temperatures during the winter months by supercooling (Sakai and Larcher, 1987). Reproductive bud tissues are isolated from acropetally advancing ice in the subtending stem by the presence of ice barriers that restrict ice growth into the meristem. Such a frost survival mechanism has been documented in reproductive buds of *R. ferrugineum* using IDTA, where single flowers within the floral bud freeze intracellularly at temperatures between -16 and -25°C, while ice formation in the stem and leaves occurs at -4°C (Neuner and Hacker, 2012). Midwinter air temperatures can drop below the supercooling temperature of overwintering reproductive buds of *R. ferrugineum* (-16 to -25°C; Larcher and Wagner, 2004) when plants are not insulated by snow cover and causes severe frost damage (personal communication, J. Wagner). Supercooling also appears to be the primary mechanism of frost survival in over-wintering reproductive buds of *Vaccinium vitis-idea* (**Figure 4**). The frost survival mechanism of reproductive overwintering buds in most other woody alpine plants has not been examined. While frost damage to reproductive buds is not critical to individual survival, it can strongly influence the reproductive success of a species. In this regard, the deleterious impact of frost injury on sexual plant reproduction has been shown to become significantly greater with increasing elevation, and at higher elevations may even impair successful seed development in most years (Ladinig et al., 2013).

## VEGETATIVE BUDS

Primordia within dormant vegetative buds have been reported to survive freezing temperatures by either extra-organ freezing or freezing tolerance (Sakai and Larcher, 1987). In vegetative buds exhibiting extra-organ freezing, ice formation does not occur in the primordial tissue itself. Water within primordial tissues remains supercooled, down to a particular species-specific and developmentally determined temperature. When freezing does occur, however, it is a lethal event. During supercooling, water migrates from the supercooled primordial tissue to specific extracellular locations in the stem and/or bud scales, thus inducing dehydrative conditions in the primordial tissues. This process has been termed extra-organ freezing (Ishikawa and Sakai, 1982). Three types of extra-organ freezing have been postulated, defined by the mode of freeze dehydration and the dehydration tolerance of bud primordia (Sakai and Larcher, 1987): In Type I, primordia are fully dehydration tolerant, become fully freeze dehydrated, and no freezable water remains. Such primordia can survive immersion in LN_2_ (-80 °C). In Type II, primordia experience freeze-induced dehydration due to the migration of water from the primordia to the remote sites of extracellular ice but have a limited level of dehydration tolerance and are thus injured at temperatures in the range of -35 to -50°C. Type III primordia become only partially dehydrated, thus freezable water remains inside the primordial cells and cells are killed when temperatures exceed the extent of supercooling (-25 and -30°C) and the primordia experience intracellular ice formation; which is a lethal event.

For woody alpine species, a paucity of information exists on the frost survival mechanism of dormant buds. Extra-organ freezing was initially reported in conifers for *Abies homolepis* and *A. balsamea* (Sakai, 1978) and appears to occur in species in the genera *Abies*, *Picea*, and *Larix* (Sakai and Larcher, 1987; Zwiazek et al., 2001). In contrast, ice seems to form innocuously in *Pinus* shoot primordia, without any large degree of supercooling, at the same time ice forms in stem tissues; with frost injury occurring at temperatures much lower than when ice formation initially occurs (Ide et al., 1998). Preliminary results on alpine conifers also suggest that similar genus-specific mechanisms of frost survival are present in their dormant vegetative buds (**Table 2**). *P. cembra* and *P. mugo* tolerate extracellular ice formation in their vegetative buds while *P. abies* and *L. decidua* seem to survive by supercooling and extra-organ freezing.

**FIGURE 5:**
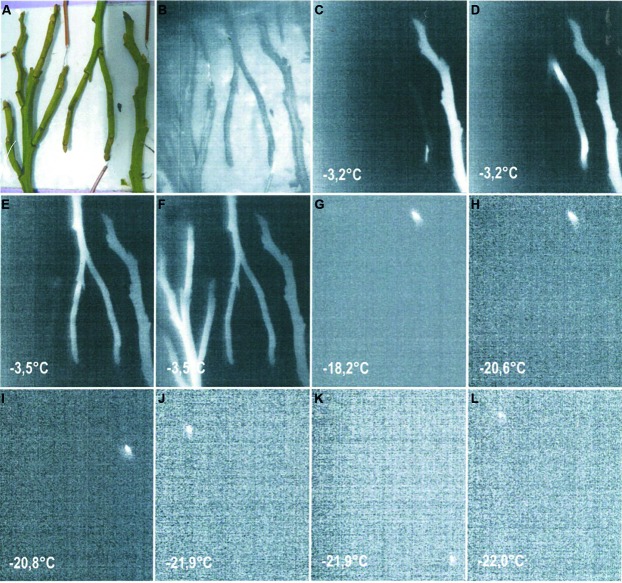
**(A)** Digital image of shoots of *Vaccinium myrtillus* sampled in winter (17.1.2014) from below a thick snow cover before exposure to a controlled freezing treatment with a cooling rate of 3 K/h. **(B–F)** IDTA images show ice propagation in the stems occurring at temperatures between -3.2 and -3.5°C. Ice propagates unhindered throughout the whole shoot. **(G–L)** Single bud meristems froze independently at temperatures ranging from -18.2 and -22.0°C, which are significantly lower than the temperature at which ice was formation was initiated in the shoot. Freezing is visualized by a brightening in the image, while unfrozen areas remain black (Miller and Neuner, unpublished).

Even less information exists for dormant buds of angiosperm species. Although information on the frost resistance of angiosperm vegetative buds is available (Sakai and Larcher, 1987), the frost survival mechanism of vegetative bud primordia is still little understood. In one study (Ishikawa et al., 1997), lateral vegetative buds of *Acer japonicum* were reported to exhibit Type I extraorgan freezing, i.e. primordia become fully dehydrated due to the loss of water to sites of extracellular ice outside of the primordial tissues and the primordia are extremely dehydration tolerant. Type II extra-organ freezing was suggested to occur in dormant apple buds (Pramsohler and Neuner, 2013). There is also a report that the vegetative buds of the angiosperm, *Pyrus syriaca*, exhibit Type III extra-organ freezing (Rajashekar and Burke, 1978). Recent observations on dormant vegetative buds of *Rhododendron ferrugineum* also indicate that they supercool (Zimmermann and Neuner, unpublished). Supercooling, as a mechanism of frost resistance, also seems to be apparent in vegetative buds of *V. myrtillus* (**Figure 5**). Based on data obtained from IDTA images, stems in winter initially froze between -3.2 and -3.5°C during a controlled freezing experiment, while buds appeared to freeze in the range of -18.2 to -22.0°C. Separate freezing events located in the resting buds can be clearly recognized. This is still a higher freezing temperature, however, than the reported midwinter frost resistance of -35°C for *V. myrtillus* buds (Sakai and Larcher, 1987). Since all of the samples used in the controlled freezing experiments were dug out from under snow, it is presumed that the moderate temperature conditions below the snow are the reason for the lower frost resistance observed in the IDTA.

Winter frost resistance of dormant buds of woody alpine shrubs ranges from -30 to -50°C (see **Table 2**) which is similar to the range found in trees, with the exception of *P. cembra* whose buds have been shown to acquire an LT_50_ of -63.6°C in midwinter, which is more cold hardy than the leaves (-58.5°C; Buchner and Neuner, 2011). In fact, the frost resistance of resting buds of conifers generally seems to be higher than that of leaves. In a global survey of maximum winter frost resistance in conifers (Bannister and Neuner, 2001) inhabiting climatic zones 7 and greater, 56% had a level of frost resistance in vegetative buds that was equal to the frost resistance of leaves, and 33% had vegetative buds that were more frost resistant than leaves. Only 11% had leaves that were more cold hardy than buds. No conclusion can be drawn, however, from the scarce data available for angiosperms.

## JUVENILE LEAVES AND SHOOTS

Young, developing juvenile leaves and shoots are typically freezing sensitive and exhibit frost damage close to the temperature at which ice formation occurs (Taschler et al., 2004; Ladinig et al., 2013). Moderate night frosts during the annual period of growth in June have been repeatedly observed to cause severe frost damage to sprouting shoots and leaves of subalpine woody species in the European Alps (Kerner, 1869; Matuszkiewicz, 1977; Christersson et al., 1987; Taschler et al., 2004; Neuner, 2007; Neuner and Beikircher, 2010). It is very clear that current year growth is highly susceptible to frost damage. Initial frost damage (LT_i_) can be observed after exposure to -3.4°C (*Rhododendron ferrugineum*), -4.4°C (*Picea abies*), -4.8°C (*Pinus cembra*), -5.5°C (*Sorbus aucuparia*), and -6.3°C (*Larix decidua*). These temperatures are likely to occur in the treeline ecotone during the annual periods of growth (Taschler et al., 2004). Only *P. cembra* seems capable of escaping natural frost damage due to a later onset of sprouting (late June to July), when potential air temperature minima are much more moderate and insufficient to cause frost injury.

Importantly, after a loss of current year’s growth, conifers do not foliate again until the next growing season. While this type of frost injury and subsequent delayed growth did not appear to threaten the survival of trees (Tranquillini, 1979; Sakai and Larcher, 1987; Gross et al., 1991), it was suggested that it may partly contribute to the distorted growth of small trees in the krummholz belt (Tranquillini, 1979).

## MATURE LEAVES

In contrast to all other plant parts, frost resistance in leaves of woody alpine species has been frequently studied. Unlike developing leaves and shoots, the frost resistance of mature leaves generally appears to exceed environmental demand. Mature leaves of alpine woody species are ice tolerant. The temperature at which initial frost damage (LT_i_) occurs in the summer is species-specific but generally occurs in the range of -4.1 to -9.0°C (**Table 2**; Taschler and Neuner, 2004). Evergreen leaves of woody species can attain a midwinter frost resistance of -26.0 to -58.5°C when cold acclimated under natural conditions. A maximum frost resistance in needles of as low as -90°C has been reported for the pine species, *P. cembra* and *P. mugo* (LT_0_; Sakai and Okada, 1971) which suggests that species that occupy the alpine region in the European Alps may have the capacity to develop a greater level of frost resistance under the proper acclimating conditions.

**FIGURE 6:**
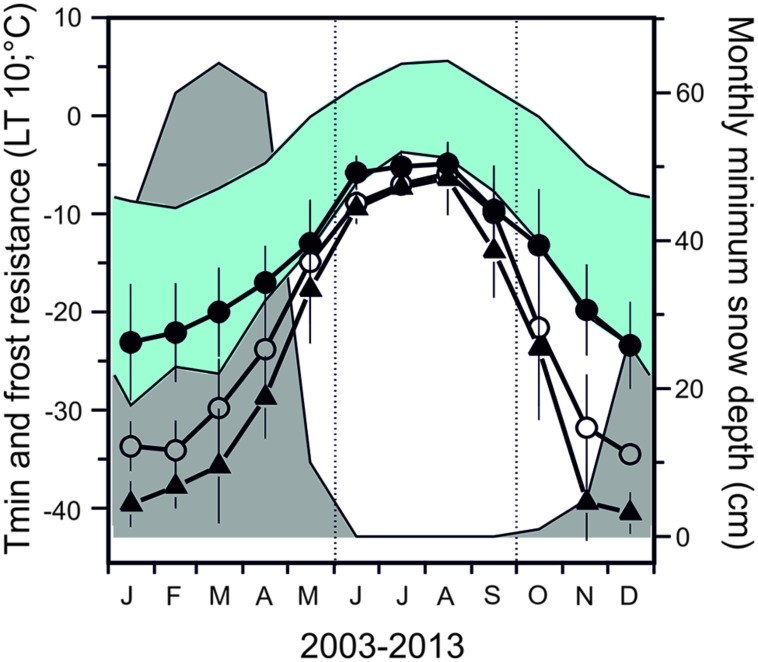
**Monthly mean values of frost resistance (LT_**10**_) of leaves of the evergreen shrub (●) *Rhododendron ferrugineum* and of the coniferous trees, (○) *Picea abies* and (△) *Pinus cembra*.** Values were calculated from data obtained throughout the entire 20^th^ century (data from: 1935/36: Ulmer, 1937; 1942/43/44: Pisek and Schiessl, 1947; 1965/66/67: Schwarz, 1970; 1993/94/95/96: Neuner et al., 1999b). Monthly mean and absolute air temperature minima (blue area) and monthly minimum of snow depth data (gray area) were obtained from a 46 year measurement period (ZAMG, Meteorological Station Mt. Patscherkofel 2247 m a.s.l.) close to the sampling site on Mt. Patscherkofel.

**FIGURE 7:**
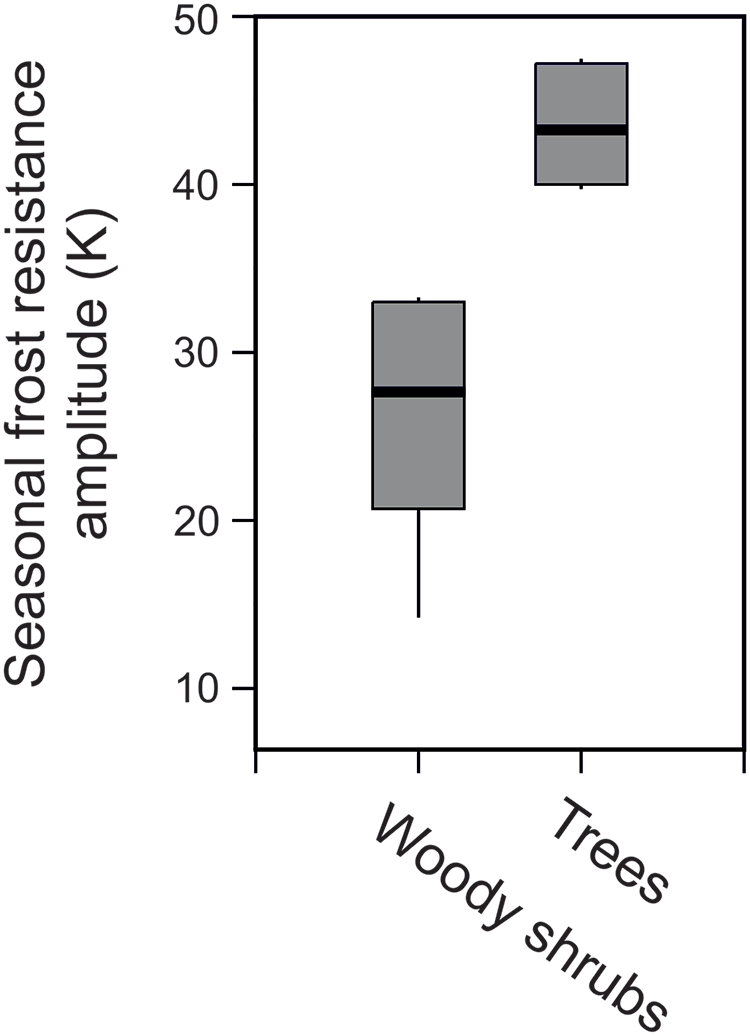
**Seasonal amplitude of frost resistance (K) in evergreen leaves of trees (*Picea abies*, *Pinus cembra*) compared to six woody alpine shrubs (*Pinus mugo*, *Arctostaphylosuva-ursi*, *Rhododendron ferrugineum*, *Vaccinium vitis-idea*, *Loiseleuria procumbens*, *Calluna vulgaris*) under natural frost hardening in the treeline ecotone on Mt. Patscherkofel**.

Seasonal data on frost resistance (LT_10_ monthly means) of leaves of the evergreen shrub *Rhododendron ferrugineum* and the coniferous trees, *Picea abies* and *Pinus cembra*, determined by various authors throughout the 20^th^ century at the same study site (Mt. Patscherkofel, 1935/36: Ulmer, 1937; 1942/43/44: Pisek and Schiessl, 1947; 1965/66/67: Schwarz, 1970; 1993/94/95/96: Neuner et al., 1999b) are shown in **Figure 6**. The seasonal amplitude of frost resistance varies in a species-specific manner. *Rhododendron* shrubs develop a lower level of midwinter freezing resistance than the coniferous trees. The seasonal amplitude of frost resistance of leaves in the evergreen trees *P. abies* and *P. cembra* is approximately 40–50 K, but in shrubs it is much less, ranging between 15 and 35 K (**Figure 7**).

In contrast to trees, smaller statured woody shrubs are usually covered by thermally insulating snow and are thus exposed to more moderate temperature conditions during the winter. Early snowfalls in autumn can interfere with the natural process of cold acclimation (Neuner et al., 1999b), reducing the maximum frost resistance attained in midwinter. Additionally, moderate temperature conditions beneath snow cover in subalpine environments have been shown to be conducive to frost dehardening (Sakai and Larcher, 1987; Neuner et al., 1999b). The maximum frost resistance of leaves of *R. ferrugineum* collected in January at the same site but in different years throughout the last century was between -14.0 and -29.5°C (LT_10_; Neuner et al., 1999b). During mild winters with early snow melt, plants do not frost harden to the same extent as when they remain exposed until midwinter (Neuner et al., 1999b). This variability in frost resistance can also be observed in leaves collected below a thick snow vs. leaves collected from exposed branches on the same sampling date and site (**Table 3**). Differences in frost resistance of up to 13 K could be observed. The most prominent environmental differences affecting exposed vs. snow-covered leaves are the severity of freezing temperatures, the irradiation climate, and plant water status. Woody plants that are exposed experience much lower freezing temperatures under natural irradiation and daylength conditions and quite often are exposed to severe winter drought. In contrast, the insulating properties of snow significantly mitigates freezing temperatures, keeping them close or slightly below 0°C. Additionally, under 15 cm of snow, PPFD can be nearly to completely eliminated and the darkness may protect plants from dehardening in response to long days in May. Additionally, a thick snow pack is the best way to escape winter drought (Sakai and Larcher, 1987). Firstly, water loss is nearly eliminated when plants are covered by a thick layer of snow and secondly, snow cover gets successively wetter during the course of winter (Kuhn, 2012) allowing to restore water deficits by direct water uptake from the wet snow.

**Table 3:** Midwinter frost resistance (LT_**50**_) measured for leaves collected at either 600 m or 1950 m, and either below a thick snow cover or from exposed shoots on 17.1.2014.

Plant species	1950 m No snow protection (LT_50_ ± SD; °C)	1950 m Snow protection (LT_50_ ± SD; °C)	600 m No snow protection (LT_50_ ± SD; °C)
*A. uva-ursi*	–41,6 ± 10,1	–34,3 ± 2,4	–
*P. abies*	–43,4 ± 6,3	–37,3 ± 5,4	–33,0 ± 4,4
*V. vitis-idea*	–42,4 ± 5,6	–29,4 ± 4,0	–23,5 ± 1,2

Similarly, frost resistance in leaves of *P. abies,* which in cold winter periods approaches -45°C at the treeline, was only -30°C during the exceptionally warm winter of 1987/88 (Körner, 2012). Furthermore, plants of the same species cold acclimate to a lesser extent at a lower elevation (*P. abies:*-10 K, *V. vitis-idea:*-19 K) compared to plants located at treeline (see **Table 3**). This corroborates the findings of Gross (1989 cited in Körner, 2012) who reported an increase in the frost resistance of *P. abies* leaves from -30°C at 950 m to -35°C at timberline in the central Alps.

The risk of frost damage changes significantly with season, which can be derived from the relationship between the temperatures representing frost resistance vs. temperature minima (see **Figure 6**). Leaves of trees at the alpine treeline have a low risk of frost damage at midwinter. This has been previously recognized, as tree-sized woody plants were suggested to exhibit a sufficient level of freezing resistance to avoid freeze damage (Larcher, 1985; Hadley and Smith, 1987; Gross et al., 1991; Perkins et al., 1991; Havranek and Tranquillini, 1995; Perkins and Adams, 1995; Körner, 2012). Regarding shrubs, given that timely snow falls provide a thermal insulation, the risk of freeze injury must be also considered rather low in winter. This may not be the case, however, if the pattern, duration, and timing of snow cover changes in future.

The overall level of risk for freezing injury in the spring is very different than during midwinter. In general, small statured woody plants have a high risk of frost damage in spring, particularly when these plants have partially deacclimated or never developed full hardiness due to snow cover, and then lose their snow cover in late winter (Sakai and Larcher, 1987; Havranek and Tranquillini, 1995; Körner, 2012). Severe night frosts can occur in the subalpine environment during this time of the year and the loss of snow cover along with their lower level of frost resistance makes them especially vulnerable to freezing injury. In such a scenario, the potential risk of frost damage will largely depend on the species-specific ability to rapidly reacclimate. The ability to reacclimate may be reduced in woody plants at this time of year due to changes in the physiology of these plants induced by their exposure to longer photoperiods (Schwarz, 1970). Little knowledge exists for most woody plant species with respect to their ability to reacclimate or the rate at which they can reacclimate.

To examine the rate of frost re-hardening (reacclimation) in spring, *R. ferrugineum* shrubs were exposed *in situ* to controlled night frosts close to the lowest temperature that did not induce frost damage (LT_0_; Neuner et al., 1999b). This experimental protocol was used to determine the potential frost re-hardening response under otherwise completely natural environmental conditions (Neuner et al., 1999b). After a lag period of three days, during which no significant increase in frost resistance was observed, frost resistance suddenly increased by 5.9°C within 24 h. Although a total gain in frost hardiness between 7.8 and 9.2°C was possible, the retarded response appeared too slow to offer sufficient frost protection from a sudden frost event. In a recent study, Wheeler et al. (2014) also reported that earlier snow-melt may increase the risk of a lethal spring freezing event for less frost-resistant alpine dwarf shrubs, such as *V. myrtillus* and *V. uliginosum*. This is in line with observations on subarctic heath that readily responds to increased temperatures by frost dehardening (deacclimation; Bokhorst et al., 2010).

Trees may also be at risk of frost damage in the spring. *In situ* exposure of *P. cembra* to increased air temperatures in late winter induced rapid deacclimation (Buchner and Neuner, 2011). Similar reports exist for other woody species that also respond to warmer spring temperatures with an untimely reduction in frost resistance (*Picea rubens*: Strimbeck et al., 1995; *P. sylvestris*: Repo et al., 1996; *Vaccinium myrtillus*: Taulavuori et al., 1997; *Betula pubescens*: Taulavuori et al., 2004). Premature frost dehardening of *P. cembra* in late winter, induced by artificially increased air or soil temperatures, reduced the frost resistance of buds to -10.2°C (LT_50_) while the untreated controls still had an LT_50_ of -32.6°C (Buchner and Neuner, 2011). During episodes of low temperature in April at the alpine timberline, air temperature minima can still drop down to -16.3°C (30 years temperature record; ZAMG). This temperature would be sufficiently low enough to cause significant frost damage to *P. cembra*. Warmer temperatures in late winter will also induce earlier bud burst in *P. cembra* (Buchner and Neuner, 2011) which must be considered a critical concern for a species that currently escapes frost damage during budbreak and the initial growth of current year shoots by delaying budbreak until the risk of significant frost events is low.

## STEMS

The bark tissue of temperate woody plants typically exhibit freezing tolerance, undergoing equilibrium freezing when ice forms in its tissues and the temperature continues to drop (Sakai and Larcher, 1987). In contrast, xylem tissues can exhibit different responses to freezing temperatures. Xylem parenchyma cells (XPC) are the only living cells in the xylem. In some species, XPCs have been shown to survive freezing temperatures by deep supercooling down to -40°C or even lower (Sakai and Larcher, 1987). With the exception of *Arctostaphylos uva-ursi,* where XPCs were reported to exhibit deep supercooling (Becwar et al., 1981), the mechanism of frost survival of xylem tissues of European alpine woody plants is not known. In a survey of woody treeline species in Colorado, the XPCs of some species in genera such as *Pinus, Picea*, *Abies,* and *Juniperus,* exhibited supercooling. This could be indicative for their European counterparts but remains to be elucidated. Freezing of water in the xylem tissue can also have secondary freezing effects that are not discussed in the present review, namely freeze-induced xylem embolism (Sperry et al., 1994).

## CONCLUSION

Various frost resistance mechanisms have evolved in alpine woody plants. In addition to tolerating the presence of ice in their tissues and the concomitant dehydrative stress, some tissues and organs that rely on freezing avoidance can also be found. In many species, the mechanism by which they survive freezing temperatures is unknown, and thus represents a fertile area for future research.

In freezing sensitive tissues, such as reproductive shoots and buds, and young developing shoots, escape from frost damage is only possible by preventing the initial formation of ice (freeze avoidance). This can be realized in woody plants by a structural ice barrier that prevents ice propagation from other tissues into the freezing sensitive tissues, and the absence or inhibition of ice nucleation active substances in the freezing sensitive tissues or organs which would allow them to supercool. The specific features of structural ice barriers still remain to be elucidated. Outside of a direct role for the absence of mature vascular tissue which would act as a conduit for the spread of ice, the determining factor(s) involved in the prevention of the spread of ice into buds and reproductive shoots is still an open question but may involve specific anatomical and biochemical (cell wall impregnation and porosity, dry tissue regions) adaptations. Additionally, hardly any attention has focused on the presence of metabolites in supercooled tissues which may facilitate their ability to remain in an ice free state.

Woody alpine species can experience frost damage throughout the whole year. In summer, the most frost susceptible organs of woody species are reproductive shoots (-4.6°C); followed by immature leaves (-5.0°C), fully expanded leaves (-6.6°C), vegetative buds (-7.3°C), and xylem tissue (-10.8°C). These levels of freezing resistance can be insufficient to survive the potential frost events that can occur in June. The severity and frequency of freezing temperatures significantly increases with increasing elevation. Perhaps it is the most frost-susceptible parts with hardly any freezing resistance that define the upper limit of elevation for the distribution of some woody species. Intriguingly, woody species that inhabit the highest elevations bury their stems in the soil, perhaps for a better protection against the impact of summer frost events.

Differences in frost resistance mechanisms and the maximum level of freezing resistance of various plant tissues and organs within the same plant stresses the importance of studying whole plants rather than extrapolating data obtained from plant parts. Most importantly, this emphasizes the need to study plants under conditions that are as close to natural as possible. Field studies that simulate frosts, or even better are conducted under natural frosts, are still rare. This can be partially attributed to the difficulties involved in conducting such studies. Their ecological significance, however, is invaluable as the whole plant; including all tissues, has an impact on the obtained results. Only the ability of plants to survive and recuperate under natural and complex environmental conditions will provide a realistic picture of the potential frost survival of a species at a certain site.

Mean frost resistance in winter is lowest in reproductive buds (-23.4°C), that without snow cover may not escape frost damage in all winters. In contrast, the freezing resistance of all other organs exhibit greater levels of freezing resistance that vary in a species-specific manner (vegetative buds: -30 to -50°C, leaves: -25 to -58.5°C, stem: -30 to -70°C). Seasonal data on frost resistance of leaves of woody alpine species at treeline under natural frost hardening indicate that a sufficiently high level of frost resistance is attained for trees in midwinter to allow them to survive naturally occurring winter freezing temperatures. Leaves of some shrubs, however, may need to rely on the thermal insulation provided by snow cover.

Regarding the risk of frost damage to woody plants, seasonality is a significant factor, particularly given the projected scenarios associated with climate change (Hänninen and Tanino, 2011). The transition period from endo- and ecodormancy in winter and early spring, to the active growing period in summer, is of special concern. Till now, the dynamics of seasonal and short-term frost hardening has been studied nearly exclusively in evergreen leaves; scarce knowledge exists for other organs and tissues. Phenological data in Europe (1971–2000) has indicated that 78% of all leafing, flowering, and fruiting records have advanced and that the average advance of spring has been 2.5 days decade^-1^ (Menzel et al., 2006). Alteration in the timing of bud burst may result in a species being less well adapted to the local temperature environment (Kramer, 1995). This includes an increased probability of spring frost damage (Murray et al., 1989; Hänninen, 1991, 1996; Kramer, 1994). Premature deacclimation (frost dehardening), once photoperiod signals the approach of spring and chilling requirements are fulfilled, is a realistic probability for alpine tree species (*P. cembra,* Buchner and Neuner, 2011). Woody shrubs, receiving thermal insulation from an early snow fall, harden to a lesser extent, and can occasionally deacclimate below the snow cover. The ability to reacclimate and the speed at which a species can reacclimate in response to unfavorable temperatures is an important parameter that remains virtually unexplored (Neuner et al., 1999b). Generally, the dynamics of the processes that fine-tune the level of frost hardiness to prevailing environmental conditions are only understood to a limited extent. If future scenarios of climate change occur as predicted by Katz and Brown (1992), where in addition to global warming, the frequency of unpredictable extreme temperature events increases, the ability of plants to reacclimate in a timely manner or resist budbreak may be even more critical in the future.

## Conflict of Interest Statement

The author declares that the research was conducted in the absence of any commercial or financial relationships that could be construed as a potential conflict of interest.
